# Uniaxial strain-induced mechanical and electronic property modulation of silicene

**DOI:** 10.1186/1556-276X-9-521

**Published:** 2014-09-22

**Authors:** Rui Qin, Wenjun Zhu, Yalin Zhang, Xiaoliang Deng

**Affiliations:** 1 National Key Laboratory of Shock Wave and Detonation Physics, Institute of Fluid Physics, No. 64, Mianshan Road, Mianyang 621900, People's Republic of China; 2 Institute of Computer Application, No. 64, Mianshan Road, Mianyang 621900, People's Republic of China

**Keywords:** Silicene, Uniaxial strain, First-principles calculation

## Abstract

We perform first-principles calculations of mechanical and electronic properties of silicene under uniaxial strains. Poisson's ratio and the rigidity of silicene show strong chirality dependence under large uniaxial strains. The ultimate strains of silicene with uniaxial strain are smaller than those with biaxial strain. We find that uniaxial strains induce Dirac point deviation from the high-symmetry points in the Brillouin zone and semimetal-metal transitions. Therefore, no bandgap opens under the uniaxial strain. Due to its peculiar structure and variable *sp*^3^/*sp*^2^ ratio of the chemical bond, the deviation directions of Dirac points from the high-symmetry points in the Brillouin zone and variation of Fermi velocities of silicene exhibit significant difference from those of graphene. Fermi velocities show strong anisotropy with respect to the wave vector directions and change slightly before the semimetal-metal transition. We also find that the work function of silicene increases monotonously with the increasing uniaxial strains.

**PACS numbers:**

61.46.-w; 62.20.D-; 73.22.Dj

## Background

Silicene, the silicon analog of graphene, is a two-dimensional honeycomb lattice of silicon atoms. It has been theoretically predicted long ago [1] but has only been synthesized recently [2-10]. Due to a similar topology with graphene, silicene has many outstanding properties like graphene, such as the massless Dirac fermion behavior [6,9] and a high Fermi velocity [9]. In addition, silicene has apparent compatibility to the current silicon-based electronic industry over and above graphene. Thus, silicene also has great potential applications in nanoelectronics and spurred much attention these years.

Recent theoretical studies demonstrated that inert substrates [11] and point defects [12] could effectively tune the electronic band structures of silicene. Meanwhile, mechanical strain often brings about astonishing effects on properties of silicon materials. It can improve the mobility of bulk Si [13,14], and strain engineering is considered to be one of the most promising strategies for developing high-performance sub-10-nm silicon devices [14]. For one-dimensional silicon nanowires, strain can tune the size of the bandgap and turn the nature of the bandgap from indirect to direct [15,16]. Since electrons of silicon atom tends to form *sp*^3^ hybridization rather than *sp*^2^ hybridization, the two-dimensional silicene can be easily buckled and have various configurations with different *sp*^3^/*sp*^2^ ratios. Freestanding silicene is predicted to favor a low-buckled configuration [1,17,18]. Silicene grown epitaxially on silver substrates in recent experiments is proposed to have several structures, such as the so-called (4 × 4) [9] or $\left(\sqrt{3}\times \sqrt{3}\right)$[8,19] structures. Theoretical investigations also suggest several different types of silicene superstructures on the Ag(111) surface [20]. The buckling gives silicene more flexibility over graphene and opens large opportunities for exploring interesting electromechanical properties in a buckled two-dimensional structure. Moreover, despite the great desire to obtain freestanding silicene, currently, silicene is mainly synthesized by epitaxial growth on substrates like Ag [5,8,9,19], ZrB_2_[7], and Ir [10]. The substrate could introduce strain, and understanding of strain effect is also fundamental for silicene growth. Recently, several groups have carried out theoretical investigation of the strain effect on the mechanical and electronic properties of silicene [21-28]. Silicene is found much less stiffer than graphene [21,27] and has a lower yielding strain [27]. Qin [27] and Liu [22] suggest that biaxial strain could induce a semimetal-metal transition. Compared with biaxial strain, uniaxial strain can further destroy the symmetry of silicene and is expected to bring new features. Zhao [28] and Mohan [24] claimed that uniaxial tensile strain can open a finite bandgap for silicene, which is important for applications in semiconductor devices. Meanwhile, Wang [29] declared that uniaxial strains will not open bandgaps for silicene and germanene. However, former studies of graphene [30-35] suggest that the uniaxial strain could introduce a new mechanism, and its effect needs to be studied cautiously.

In this work, we perform a careful and systematic first-principles study of the uniaxial strain effect on the mechanical and electronic properties of silicene. We find that Poisson's ratio and rigidity of silicene show strong chirality dependence, and the ultimate strain of silicene under uniaxial strain is smaller than that under biaxial strain. We show that no bandgap opens in silicene under uniaxial strain in the elastic region. Uniaxial strains shift the Dirac cones from the high-symmetry points in the Brillouin zone and induce a semimetal-metal transition for silicene. Variation of the band structures exhibits strong anisotropy. Fermi velocity under uniaxial strain is found to be a strong function of the wave vector and changes slightly before the semimetal-metal transition. The work function increases significantly under uniaxial strains.

## Methods

**Figure 1:**
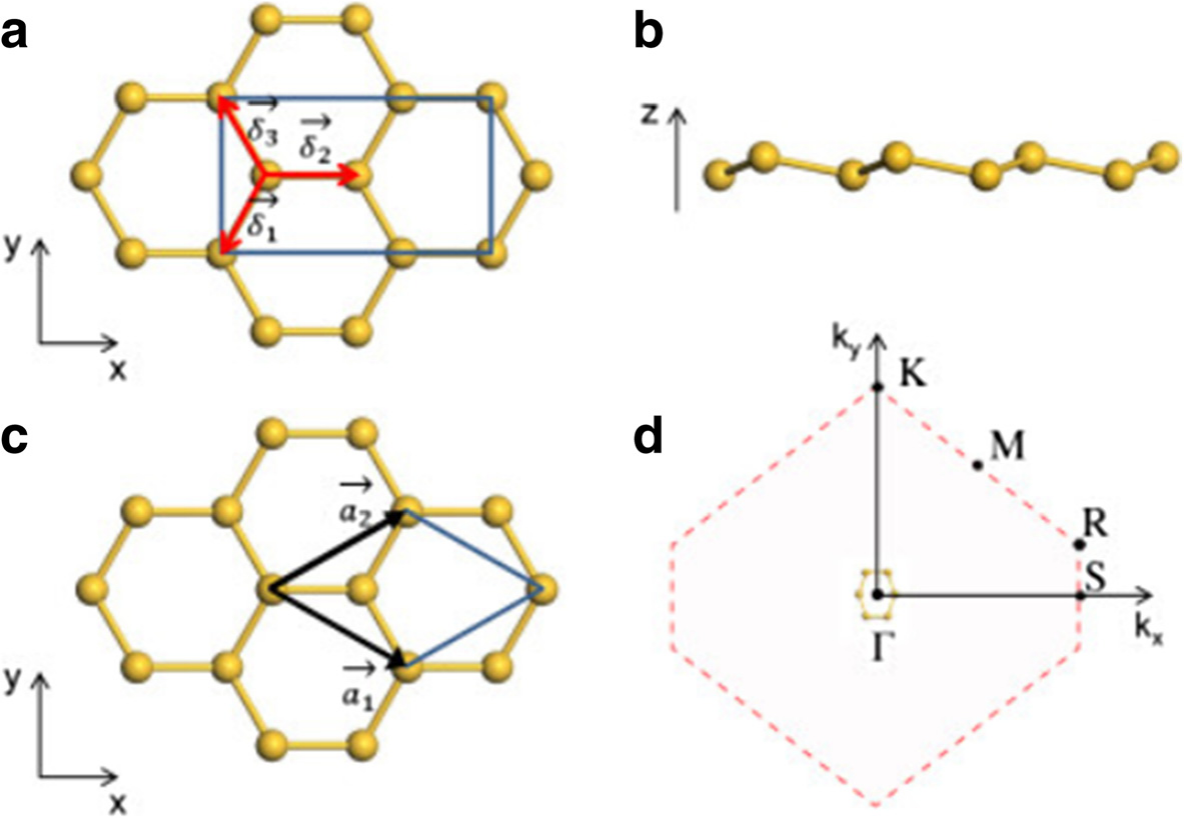
**Silicene configuration and its first Brillouin zone. (a)** Top view of the four-atom rectangular unit cell of silicene. ***δ***_*i*_ (*i* = 1, 2, and 3) are the three nearest-neighbor connecting vectors. **(b)** Side view of silicene. **(c)** Top view of the two-atom primitive cell of silicene. ***a***_1_ and ***a***_2_ are the lattice vectors. ***a***_1_ = (*a*_*x*_, -*a*_*y*_) and ***a***_2_ = (*a*_*x*_, *a*_*y*_). **(d)** Schematic of silicene under the ZZ strain and the corresponding first Brillouin zone with high-symmetry points.

The low-buckled configuration of silicene is studied by using a periodic supercell model. A vacuum of nearly 20 Å is chosen to eliminate the interactions between adjacent silicene sheets. The first-principles calculations are performed within the density functional theory (DFT) implemented in the DMol^3^ package [36,37]. The local density approximation (LDA) is employed for the exchange-correlation functional. All electron treatment and double numerical plus polarization basis sets are applied for silicon atoms. A 40 × 40 × 1 Monkhorst-Pack *k*-point mesh [38] for the Brillouin zone sampling is used for the 1 × 1 hexagonal supercell which contains two silicon atoms (Figure 1c), and this sampling is scaled according to the size of the supercells in our calculations. The structures are optimized until the maximum force allowed on each atom is less than 10^-4^ eV/Å.

## Results and discussion

**Figure 2:**
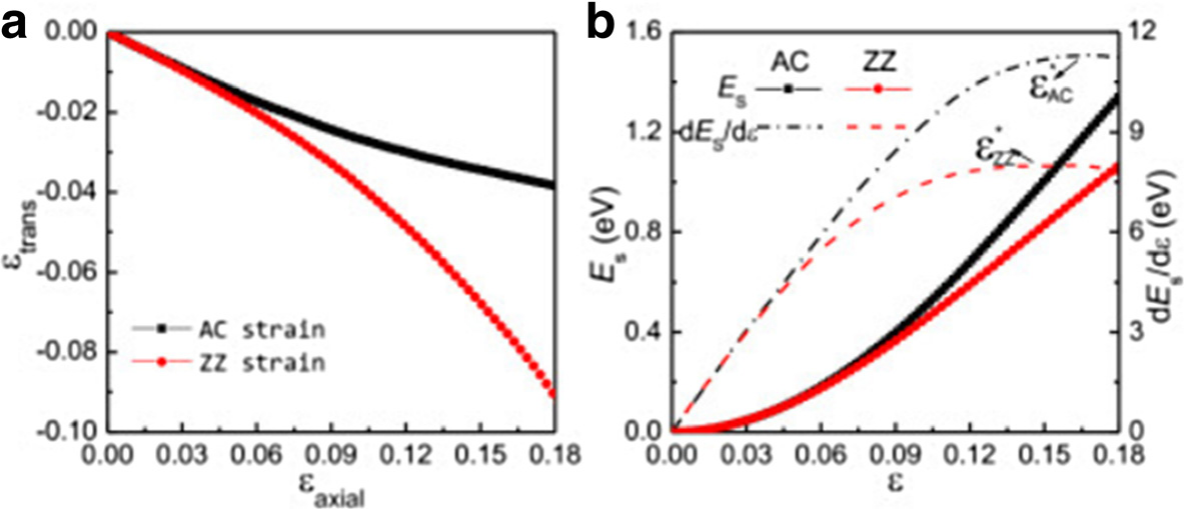
**Transverse strain and strain dependencies. (a)** Transverse strain (*ϵ*_trans_) as a function of the axial strain (*ϵ*_axial_) for axial strains along the AC and ZZ directions of silicene. **(b)** Strain dependencies of the strain energy (*E*_S_) and its derivative (d*E*_S_/d*ϵ*) of silicene under AC and ZZ strains. ϵ^*^_AC_ and ϵ^*^_ZZ_ denote ultimate strains of silicene under AC and ZZ strains, respectively.

First, we relax the silicene structure in the absence of strain. The lattice constant of the hexagonal lattice, buckling distance, and Si-Si bond length of the relaxed silicene are found to be 3.83, 0.45, and 2.25 Å, respectively, which are in good agreement with previous calculations [18]. Then, we investigate the effect of uniaxial strains along two special directions of silicene: the armchair, AC, (*x* axis in Figure 1a) and zigzag, ZZ, (*y* axis in Figure 1a) directions. A rectangular silicene supercell (Figure 1a) is constructed to study the uniaxial strain effect. In our calculations, strain *ϵ* is defined as *ϵ* = (*a* - *a*_0_)/*a*_0_, where *a* and *a*_0_ are lattice constants with and without strain, respectively. AC and ZZ strains up to 0.18 are applied by gradually increasing the corresponding axial lattice constants, while the transverse lattice constants are optimized to relax the other stress components. For axial strains (*ϵ*_axial_) along AC and ZZ directions, the resulting transverse strains (*ϵ*_trans_) are shown in Figure 2a as functions of *ϵ*_axial_. Due to Poisson's effect, the lattice shrinks in the transverse direction when uniaxial tension strain is applied. When 0 < *ϵ*_axial_ < 0.02, *ϵ*_trans_ decreases linearly with the increasing *ϵ*_axial_ with the same slope for both AC and ZZ strains, showing isotropy of the hexagonal lattice under small strain [39]. Poisson's ratio *v* can be directly calculated by its definitions *v* = -*ϵ*_trans_/*ϵ*_axial_ and *v*_AC_ = *v*_ZZ_ = 0.31 for small strains, which agree well with previous calculations [21,27]. When *ϵ*_axial_ > 0.2, *ϵ*_trans_ decreases nonlinearly with the increasing *ϵ*_axial_ and behaves quite differently for the AC and ZZ strains, indicating that silicene is anisotropic under large strain. When *ϵ*_axial_ increases, *ϵ*_trans_ for the AC strain decreases more and more slowly, while *ϵ*_trans_ for the ZZ strain decreases more and more rapidly. Thus for large strains, Poisson's ratio decreases for the AC strain and increases for the ZZ strain with the increasing strain.

We also calculate the strain energy to investigate the mechanical response of silicene under uniaxial strain. Here, the strain energy *E*_S_ is defined as the energy difference between systems with and without strain. The strain dependencies of *E*_S_ and its derivative d*E*_S_/dϵ are shown in Figure 2b. Over the strain range considered, *E*_S_ increases with the increasing strain, while the system keeps the similar structure and remains in the elastic region. For small strain, the *E*_S_ and d*E*_S_/d*ϵ* curves coincide for both types of uniaxial strains, and d*E*_S_/d*ϵ* increases linearly with respect to the strain. The linear behavior of the d*E*_S_/d*ϵ* curve indicates that the system is in the harmonic region. When the system goes into the anharmonic region, d*E*_S_/d*ϵ* changes nonlinearly and shows anisotropy for the AC and ZZ strains. In this region, d*E*_S_/d*ϵ* increases more quickly under the AC strain than under the ZZ strain. Thus, silicene is more rigid under stretch along the AC direction than along the ZZ direction, which is analogous with graphene [40,41]. The d*E*_S_/d*ϵ* curves have maxima at large strains for both types of strains. The strain corresponding to the maximum d*E*_S_/d*ϵ* is the ultimate strain (ϵ^*^). For strain larger than ϵ^*^, imaginary phonon frequencies are expected to appear for specific wave vectors of acoustic waves, and the system will become metastable. This phenomenon is the so-called ‘phonon instability’ [40,42,43]. The ultimate strains for the AC and ZZ strains are 0.17 and 0.15, respectively. Both ultimate strains are slightly smaller than that of biaxial strain of 0.18 [27].

**Figure 3:**
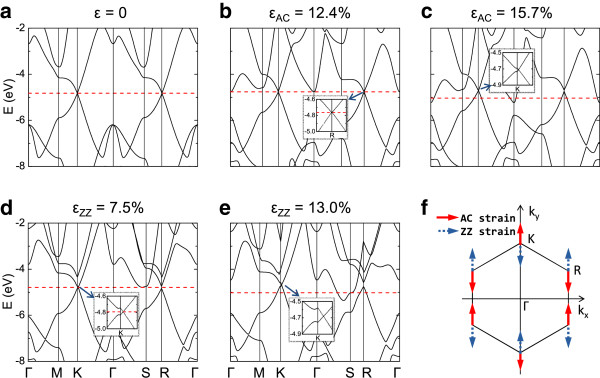
**Band structures and shift directions of Dirac points of silicene.** Band structures of silicene: **(a)** without strain, under AC strains of **(b)** 12.4% and **(c)** 15.7%, and under ZZ strains of **(d)** 7.5%, and **(e)** 13.0%, respectively. The red dashed line denotes the Fermi level. Insets: magnified portion of band structures near the Dirac point. **(f)** Shift directions of Dirac points of silicene under small tensile strains.

We then investigate the electronic properties of silicene under uniaxial strains. First, we calculate the band structures of silicene under uniaxial strains of different types and magnitudes. The hexagonal lattice is chosen to provide a Brillouin zone with well-defined high-symmetry points (Figure 1c). With uniaxial strain, the symmetries of the silicene lattice and the Brillouin zone are lowered, and the Brillouin zone is no longer a regular hexagon. The new high-symmetry points of the Brillouin zone are shown in Figure 1d. In the calculations, we use fine *k*-point samplings of over 1,800 points for the *k*-point paths to give detailed band structures. We find that without strain, the Dirac points coincide with the high-symmetry K and R points of the first Brillouin zone. With both types of uniaxial strains, the Dirac points begin to deviate from K and R in the reciprocal space while the Dirac cone remains at the Fermi level (Figure 3b,d). Like in the biaxial strain case [22,27], the σ^*^ orbitals are also sensitive to the uniaxial strains: the energy levels of σ^*^ orbitals at the Γ point and near the S point shift down rapidly toward the Fermi level with the increasing AC and ZZ strains, respectively. When the strain is large enough, the energy level of the σ^*^ orbital will eventually fall below the Fermi level, and silicene transforms from semimetal to metal. We find that the semimetal-metal transition strains for AC and ZZ strains are 12.4% and 7.5%, respectively (Figure 3b,d). The transition strain for the AC strain is much larger than those for ZZ and biaxial strain (7%) [27]. Since this transition is expected to introduce significant resistance changes at low bias voltages, the large difference between transition strains can be used to identify the strain type. In our calculations, no bandgap opens with either AC or ZZ tensile strain in the elastic region because of the Dirac cone shift and semimetal-metal transition, which agrees with Wang's results [29]. The spurious uniaxial strain-induced bandgap reported by Zhao [28] and Mohan [24] may be due to the missing of the Dirac cone deviation or misinterpreting of the band structures.

**Figure 4:**
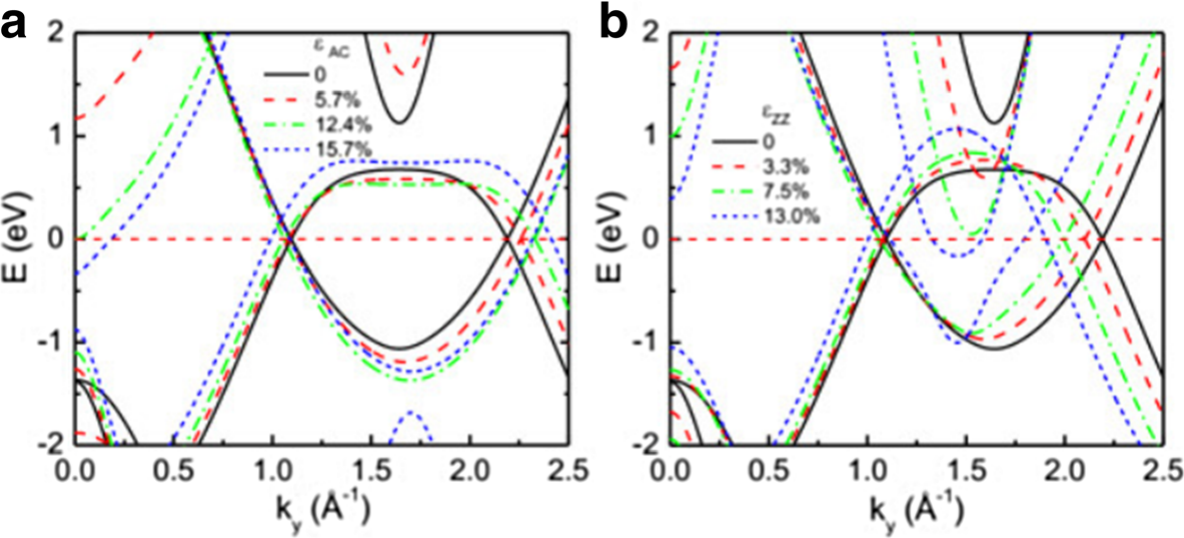
**Band structures along the line of *k***_***x ***_**= 0 in the reciprocal space (see Figure**1**d).** For various **(a)** AC and **(b)** ZZ strains, respectively. The Fermi levels are set to zero.

We find that the shift direction of the Dirac point depends on the direction and magnitude of the uniaxial strain. Since the shifting of the Dirac point from the K point is exactly opposite to that from the R point, hereafter, we only mention the former shifting. With the AC strain smaller than 13.3%, the Dirac point shifts away from the Γ point along the ΓK line (Figure 3f). When *ϵ*_AC_ > 13.3%, the Dirac point shifts in the opposite direction along the ΓK line. For the ZZ strain, the Dirac point always shifts toward the Γ point along the ΓK line (Figure 3f). Thus, with small uniaxial strains, the shift directions of the Dirac point of silicene is completely opposite to those of graphene despite their similar structures [35]. The deviation of the Dirac points from the high-symmetry points in the Brillouin zone can be understood by the tight-binding model [32,34,35]. If we only consider the nearest-neighbor hoppings, the tight-binding Hamiltonian of silicene can be written as $H=-t_{2}\sum_{k}\left[\xi \left(k\right)c_{Ak}^{†}c_{Bk}+c.c\text{.}\right]$, where *t*_*i*_ are hopping parameters depending on the nearest-neighbor connecting vectors ***δ***_*i*_ (*i* = 1, 2, 3) (Figure 1a), $\xi \left(k\right)=e^{k•\delta_{2}}\left(1\;+\;2\eta e^{-ik_{x}a_{x}}\cos\left(k_{y}a_{y}\right)\right)$, *η* ≡ *t*_1_/*t*_2_, and *c*^†^_A(B) ***k***_ and *c*_A(B) ***k***_ are creation and annihilation operators for an electron with momentum ***k*** on the sublattice A(B), respectively. The energy dispersion is obtained as *E*_***k***_ = ±*t*_2_|ξ(***k***)|. The Dirac point (0, *k*_DP_) corresponds to the point satisfying *E*_***k***_ = 0, and the solution gives $k_{\mathrm{DP}}=\frac{1}{a_{y}}\cos^{-1}\left(-\frac{1}{2\eta }\right)$[34]. The position of the K point (0, *k*_K_) can be obtained from the lattice vectors (Figure 1c), and $k_{K}=\frac{\pi }{2a_{y}}\left(1+\frac{a_{y}^{2}}{a_{x}^{2}}\right)$. Thus, $\frac{k_{\mathrm{DP}}}{k_{K}}=\frac{2\cos^{-1}\left(-\frac{1}{2\eta }\right)}{\pi \left(1+\frac{a_{y}^{2}}{a_{x}^{2}}\right)}$, and *k*_DP_/*k*_K_ = 1 when no strain is applied. The uniaxial strain not only changes the lattice structures but also modifies the hopping parameters. Under the AC strain, *a*_*y*_/*a*_*x*_ decreases due to Poisson's effect, and *t*_1_/*t*_2_ increases. Meanwhile, the buckling distance also decreases to release strain, and the proportion of *sp*^2^ hybridization in the mixture of the *sp*^3^ and *sp*^2^ hybridization of the chemical bond increases. Since the bond length corresponding to *t*_1_ is smaller than that of *t*_2_ with the AC strain, the increase of the proportion of *sp*^2^ hybridization tends to lower *t*_1_/*t*_2_ and thus enhances *k*_DP_. Eventually, *k*_DP_ > *k*_K_ for the AC strain. Similarly, the variation of the *sp*^3^/*sp*^2^ ratio leads to *k*_DP_ < *k*_K_ in the ZZ strain case. We also calculate the band structures along the line of *k*_*x*_ = 0 for AC and ZZ strains. We find that the two Dirac points along the *k*_*x*_ = 0 line repel each other with the increasing AC strain (Figure 4a) and approach each other with the increasing ZZ strain (Figure 4b). The variation of the distance between the two Dirac points along the *k*_*x*_ = 0 line is similar to that of graphene [34]. Previous calculations show that the mergence of the two Dirac points could induce a bandgap in graphene [32,34]. Nevertheless, we notice that the two Dirac points of silicene do not merge together with the ZZ strain in the elastic region, and silicene does not have the chance to open a bandgap by such a mergence.

**Figure 5:**
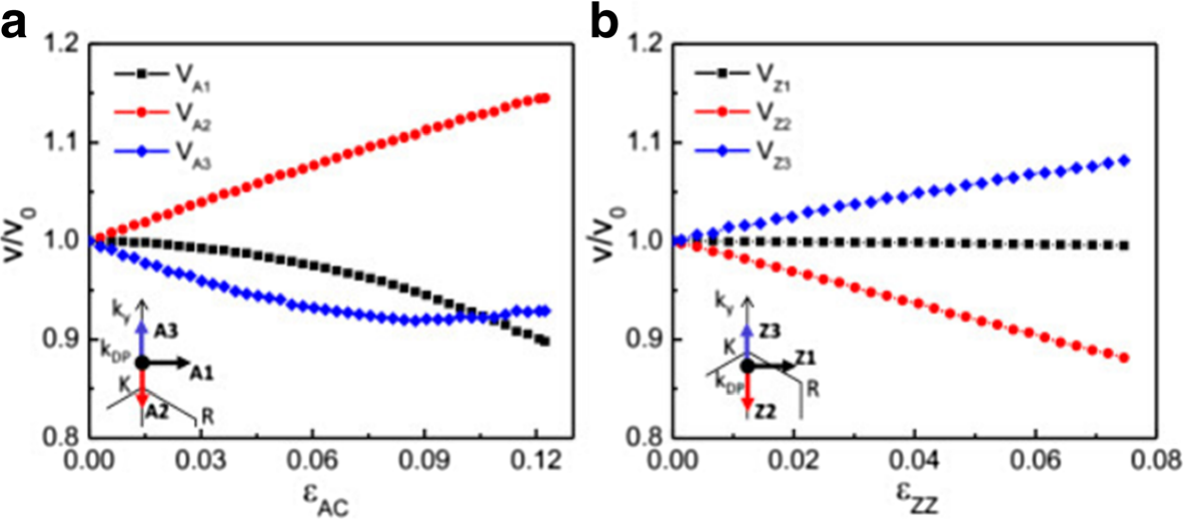
**Fermi velocities of silicene along different directions.** Under **(a)** AC and **(b)** ZZ strains in a unit of the Fermi velocity without strain (*v*_0_), respectively. Insets: different directions in the Brillouin zone. ***k***_DP_ denotes the Dirac point.

A high Fermi velocity (*v*_F_) is very important for high-speed electronic device applications. Our previous study [27] shows that the Fermi velocity of silicene is comparable to that of graphene in the absence of strain. Under the uniaxial strain, Fermi velocity also depends on the wave vector direction. We calculate the Fermi velocities of silicene along different directions for AC and ZZ strains smaller than the semimetal-metal transition strain (Figure 5). *v*_F_ is obtained by fitting the *π* and *π*^∗^ bands near the Dirac point (***k***_DP_) with the expression *E*(***q***) = *v*_F_ ħ| ***q*** |, where ***q*** = ***k*** - ***k***_DP_. With the increasing AC strain, Fermi velocity along the AC strain (*v*_A1_) decreases, and those in directions perpendicular to the AC strain behave quite differently (Figure 5a). *v*_F_ along the -*k*_*y*_ direction (*v*_A2_) increases nearly linearly with the increasing strain; *v*_F_ along +*k*_*y*_ direction (*v*_A3_) first decreases by 8.1% of *v*_F_ without strain (*v*_0_), then increases slightly with the increasing strain. Up to an AC strain of 12.2%, *v*_A2_ increases by 14.5% of *v*_0_, while *v*_A1_ and *v*_A3_ decrease by 10.2% and 7.1% of *v*_0_, respectively. With the increasing ZZ strain, Fermi velocity perpendicular to the ZZ strain (*v*_Z1_) remains nearly unchanged. Fermi velocities parallel to the ZZ strain (*v*_Z2_ and *v*_Z3_) (Figure 5b) also differ from each other: the former decreases linearly with the increasing strain, while the latter increases linearly. Up to a ZZ strain of 7.5%, *v*_Z2_ decreases by 11.9% of *v*_0_, and *v*_Z3_ increases by 8.2% of *v*_0_. A previous study shows that the next-nearest-neighbor hopping gives rise to a tilted Dirac cone in the *k*_*y*_ direction for graphene, and thus, *v*_A(Z)2_ ≠ *v*_A(Z)3_[44]. Since the Si-Si bond is longer than the C-C bond, the next-nearest-neighbor hopping plays a more important role in the low-energy properties of silicene and leads to a larger Fermi velocity anisotropy of silicene under uniaxial strain than that of graphene [34]. The significant Fermi velocity anisotropy is expected to induce the resistance anisotropy along different directions for silicene under uniaxial strain.

**Figure 6:**
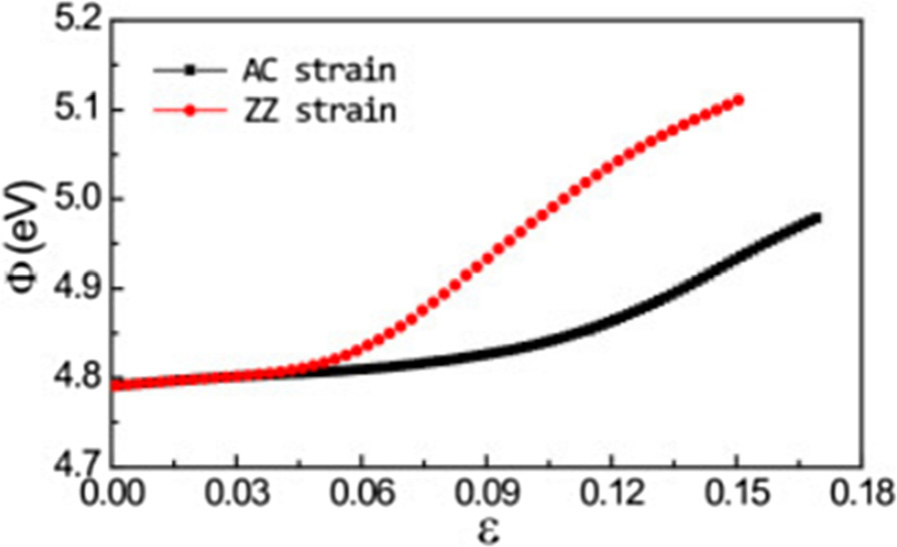
**Work function ( *Φ *) of silicene under AC and ZZ strains.**

We also investigate the work function, *Φ*, of silicene under the AC and ZZ strains (Figure 6). Work function is defined as *Φ* = *E*_vac_ - *E*_*f*_, where the vacuum level *E*_vac_ is the potential energy at infinite distance away from the material and *E*_*f*_ is the Fermi level. In our calculations, we average the electrostatic potential in the plane parallel to the silicene plane at different distances to the silicene plane. With the increasing distance to the silicene plane, the average electrostatic potential will flatten to an asymptotic value. *E*_vac_ is determined as the asymptotic value of this average electrostatic potential energy, and then the work function can be obtained by using its definition. The calculated work function of silicene without strain is 4.79 eV. When strain increases, the work function increases monotonously in the whole elastic region for both AC and ZZ strains and does not saturate at large strains like in the biaxial strain case [27]. Up to a strain of 3%, the work function increases slightly by 0.01 eV regardless of the directions of strains. Then, the work function of silicene under the ZZ strain increases more than that under the AC strain. When AC and ZZ strains reach their ultimate strains, the work functions rise up to 4.98 and 5.11 eV, respectively. The variation of the work function can be understood from the change of the Fermi level. Due to Poisson's effect, one of the nearest-neighbor distance, *δ*_2_, decreases while the other two, *δ*_1_ and *δ*_3_, increase with the increasing tensile ZZ strain. With the increasing tensile AC strain, all three nearest-neighbor distances increase. Thus, the orbital interaction with the nearest neighbors is stronger under the ZZ strain than under the AC strain, and the Fermi level decreases more rapidly under the ZZ strain than under the AC strain. Especially, the semimetal-metal transition strain for the ZZ strain (7.5%) is smaller than that for the AC strain (12.4%). After the transition, the energy levels of σ^*^ orbitals fall below the energy levels of Dirac points and become occupied, and the Fermi level falls rapidly. Therefore, the work function increases more rapidly under the ZZ strain than under the AC strain. For large strain magnitude, silicene under the ZZ strain becomes weakly coupled silicon dimmers, while silicene under the AC strain becomes weakly coupled zigzag silicon chains. Thus, work functions under ZZ and AC strains will not converge to the same value. Our calculation indicates that uniaxial strain can be used to control the band lineup in silicene-related contacts, which is important for nanoelectronic applications. In addition, uniaxial strain modulation is also useful in gas sensor applications by affecting the charge transfer between gaseous molecules and silicene.

## Conclusions

We theoretically study the mechanical and electronic properties of silicene under uniaxial strain. We find significant chirality effect on the mechanical and electronic properties of silicene. Our calculation shows that silicene remains gapless with uniaxial strain due to the Dirac point deviation and semimetal-metal transition. We find that the geometric structure and variable *sp*^3^/*sp*^2^ ratio of the chemical bond of silicene give rise to peculiar electronic properties of silicene under uniaxial strain, which are much different from those of graphene. The high Fermi velocity under uniaxial strain and strain tunable work function could promote potential applications of silicene in nanoelectronic devices.

## Competing interests

The authors declare that they have no competing interests.

## Authors’ contributions

RQ was the principal investigator of this study. RQ did most of the investigation on the mechanical and electronic properties of silicene. WZ investigated part of the mechanical properties. YZ investigated the work function. XD did part of the mechanical properties analysis. All authors read and approved the final manuscript.
